# Detection and Quantification of Citrullinated Chemokines

**DOI:** 10.1371/journal.pone.0028976

**Published:** 2011-12-16

**Authors:** Eva A. V. Moelants, Jo Van Damme, Paul Proost

**Affiliations:** Laboratory of Molecular Immunology, Rega Institute, K.U. Leuven, Leuven, Belgium; French National Centre for Scientific Research, France

## Abstract

**Background:**

Posttranslational deimination or citrullination by peptidylarginine deiminases (PAD) regulates the biological function of proteins and may be involved in the development of autoimmune diseases such as rheumatoid arthritis and multiple sclerosis. This posttranslational modification of arginine was recently discovered on inflammatory chemokines including CXCL8 and CXCL10, and significantly reduced their biological activity. To evaluate the importance of these modified chemokines in patients, methods for the detection and quantification of citrullinated chemokines are needed. Since citrullination only results in an increase of the protein mass with one mass unit and the loss of one positive charge, selective biochemical detection is difficult. Therefore, we developed an antibody-based method to specifically detect and quantify citrullination on a protein of interest.

**Methodology/Principal Findings:**

First, the citrullinated proteins were chemically modified with antipyrine and 2,3-butanedione at low pH. Such selectively modified citrullines were subsequently detected and quantified by specific antibodies raised against a modified citrulline-containing peptide. The specificity of this two-step procedure was validated for citrullinated CXCL8 ([Cit^5^]CXCL8). Specific detection of [Cit^5^]CXCL8 concentrations between 1 and 50 ng/ml was possible, also in complex samples containing an excess of contaminating proteins. This novel detection method was used to evaluate the effect of lipopolysaccharide (LPS) on the citrullination of inflammatory chemokines induced in peripheral blood mononuclear cells (PBMCs) and granulocytes. LPS had no significant effect on the induction of CXCL8 citrullination in human PBMCs and granulocytes. However, granulocytes, known to contain PAD, were essential for the production of significant amounts of [Cit^5^]CXCL8.

**Conclusion/Significance:**

The newly developed antibody-based method to specifically detect and quantify chemically modified citrullinated proteins is proven to be effective. This study furthermore demonstrates that granulocytes were essential to obtain significant levels of [Cit^5^]CXCL8. For human PBMCs and granulocytes stimulation with LPS did not affect the citrullination of CXCL8.

## Introduction

Chemokines are small chemotactic cytokines playing a role in leukocyte recruitment during leukocyte homeostasis and inflammation, in tumor development and in angiogenesis [1]. The biological activity of these proteins depends on the interaction with seven transmembrane spanning G protein-coupled receptors (GPCR), i.e. CXC and CC chemokine receptors (CXCR and CCR) [2], [3]. In addition, chemokines bind matrix- or cell-associated glycosaminoglycans [4]. The cytokine and chemokine activity is regulated at multiple levels including posttranslational modification (PTM) [5], [6]. NH_2_- and COOH-terminal proteolytic processing and glycosylation have been detected on chemokines [6]. Depending on the chemokine and on the type of PTM, reduced or enhanced receptor affinity or specificity and chemokine activity have been reported [7].

In addition to truncation and glycosylation, deimination of arginine (Arg) to citrulline (Cit) or citrullination is a recently discovered PTM on chemokines [8], [9]. The enzymes responsible for the conversion of peptidylarginine to peptidylcitrulline are peptidylarginine deiminases (PAD) [10]. Citrullination of proteins may seriously influence the organization of the protein structure and interactions in macromolecules, resulting in altered protein folding [11], [12]. Moreover, citrullinated proteins and autoantibodies to citrullinated peptides have been implicated in autoimmune diseases such as rheumatoid arthritis [13], [14] and multiple sclerosis [15].

Natural citrullination of the inflammatory chemokines interleukin-8 (IL-8/CXCL8) and interferon-induced protein-10 (IP-10/CXCL10) occurs specifically on arginine residue 5 [8], [9]. In contrast, multiple arginines (arginine 8, 12, 20, 41 and 47) in stromal cell-derived factor-1α (SDF-1α/CXCL12) are rapidly deiminated upon incubation with PAD [16]. For the chemokines epithelial cell-derived neutrophil-activating protein-78 (ENA-78/CXCL5), CXCL8, CXCL10, interferon T cell α-chemoattractant (I-TAC/CXCL11) and CXCL12, it is shown that citrullination significantly alters the biological activity [8], [9], [16]–[18]. Indeed, several protein-protein interactions, e.g. chemokine-enzyme or chemokine-receptor, and protein-glycosaminoglycans (GAG) interactions, such as heparin and heparan-sulphate, are affected. Although citrullination of CXCL8 has a minor effect on its affinity for CXCR1 or CXCR2, the binding of citrullinated CXCL8 to erythrocytes expressing the atypic Duffy antigen/receptor for chemokines (DARC) is significantly weakened [8], [18]. For CXCL12, citrullination of arginine 8 only, results in significantly reduced affinity for CXCR4, but not for CXCR7. However further citrullination reduces and even impedes the CXCR4 and CXCR7-binding capacities [16]. Deimination of CXCL10 and CXCL11 hardly affects their affinity for CXCR3 or CXCR7 [9]. The chemokine-GAG interactions are reduced upon citrullination of CXCL8, CXCL10 and CXCL11 [8], [9]. Moreover citrullination of intact CXCL8(1–77) protects this chemokine from being cleaved by the serine proteases thrombin and plasmin into the more potent 72 amino acid form [8]. All these weakened molecular interactions result in reduced *in vitro* activity (e.g. signalling, adhesion molecule expression, chemotaxis) for CXCL5, CXCL8, CXCL10, CXCL11 and CXCL12. More strickingly, citrullinated CXCL8 is unable to induce neutrophil extravasation to the peritoneal cavity *in vivo*. These studies clearly show the biological importance of the citrullination of chemokines.

Inflammatory chemokines are primarily produced upon stimulation of cells with cytokines or microbial products such as Toll-like receptor (TLR) ligands [2]. CXCL8, the main human neutrophil chemotactic protein, is produced in large amounts by neutrophils and peripheral blood mononuclear cells (PBMCs) upon bacterial infection [19]–[21]. However, no information is available on the levels of citrullinated chemokine produced by these cells.

PTMs such as NH_2_- and COOH-terminal truncations and glycosylation of chemokines which result in a significant alteration of the molecular mass were already successfully identified by mass spectrometry [22], [23]. However, citrullination of chemokines and cytokines results in an increase of only one mass unit and the loss of one positive charge. This minor change in relative molecular mass (M_r_) is difficult to detect by mass spectrometry. Here we describe a technique for the specific chemical labeling, immunobiological detection and quantification of citrullinated CXCL8 ([Cit^5^]CXCL8). Citrullinated proteins are detected and quantified by means of antibodies raised against this chemically modified citrulline. To investigate whether TLR ligands and the presence of granulocytes also influence the citrullination of inflammatory chemokines, we evaluated the absolute amounts of [Cit^5^]CXCL8 in human PBMCs and granulocytes upon stimulation with an important TLR ligand, i.e. lipopolysaccharide (LPS).

## Materials and Methods

### Ethics statement

No specific approval from an institutional review board is required for the use of buffy coats for the following reasons: (1) no personal patient information is made available, (2) buffy coats cannot be used for treatment of patients and are therefore waste products for the blood transfusion centre and (3) blood donors sign an agreement that parts of the donation that cannot be used for patient treatment may be used for scientific research.

### Reagents and materials

Bacterial LPS, antipyrine, 2,3-butanedione (>99.4% [GC]) and keyhole limpet hemocyanin (KLH) were obtained from Sigma-Aldrich (St Louis, MO, USA) and 1-ethyl-3-(3-dimethylaminopropyl) carbodiimide (EDC) from Pierce (Rockford, IL, USA). Fetal bovine serum (FBS) was purchased from HyClone (Rockford, IL, USA) and RPMI 1640 medium from Lonza (Basel, Switzerland). Samples of human blood, serum and plasma were obtained from healthy volunteers. Chemokines (CXCL8, [Cit^5^]CXCL8 and [Cit^9^]CXCL5) were chemically synthesized in-house by solid phase peptide synthesis [17], [24].

### Synthesis and chemical modification of citrulline-containing peptides

Peptides were chemically synthesized based on fluorenyl methoxycarbonyl (Fmoc) chemistry using a 433A solid phase peptide synthesizer (Applied Biosystems, Foster City, CA, USA), as described by Loos *et al.* [24]. Following synthesis, the resin and the side chain protecting groups were cleaved from the synthetic peptides with a mixture of 9.5 ml trifluoroacetic acid (TFA) and 0.5 ml water for 90 min at room temperature. The resin particles were removed by filtering the TFA solution through a Biospin filter (Bio-Rad laboratories, Hercules, CA, USA). The peptides in the filtrate were precipitated and washed with cold diethyl ether, dissolved in water, lyophilized, re-dissolved in 0.1% (v/v) TFA and purified by RP-HPLC on a 4.6×150 mm Source 5RPC column (GE Healthcare, Diegem, Belgium) applying an acetonitrile gradient in 0.1% TFA. UV absorption was monitored at 220 nm. Part of the column effluent (0.7%) was analyzed by online ion trap mass spectrometry (Esquire LC; Bruker Daltonics, Bremen, Germany). HPLC fractions containing the synthetic peptides were lyophilized and pooled in ultrapure water and chemically modified by adding 25 mM antipyrine, 16% TFA and 6.25 mM 2,3-butanedione, as previously described [25], [26]. The mixture was incubated for 2 h at 37°C in the dark, diluted with ultrapure water and repurified on a 4.6×150 mm Source 5RPC column applying an acetonitrile gradient in 0.1% TFA and detected at 220 nm. Part of the column effluent (0.7%) was analyzed by online ion trap mass spectrometry (Esquire LC, Bruker Daltonics). Fractions containing the peptide with correct M_r_ were pooled, lyophilized and dissolved in ultrapure water (MilliQ; Millipore, Billerica, MA, USA). The peptide sequences and concentrations were determined by Edman degradation on a 491 Procise cLC protein sequencer (Applied Biosystems).

### Antibody production and purification

2 mg chemically modified synthetic peptide and 2 mg KLH were dissolved in 50 mM MES (2-[*N*-morfolino]ethane sulfonic acid) buffer (pH 5). 50 µL of 50 mM EDC was added and the resulting mixture incubated for 2 h at room temperature. The immunogen was injected intramuscularly in a New Zealand white rabbit (*Oryctolagus cuniculus*) and blood samples were collected from the ear veins 8 to 20 days after immunization. Serum was filtered and antibodies were purified by affinity chromatography on a Protein G Sepharose column (GE Healthcare). Collected elutions were analyzed by enzyme-linked immunosorbent assay (ELISA) to verify the presence of specific antibodies against chemically modified citrulline residues.

### Detection of total CXCL8

The level of total human CXCL8 was quantified by a specific sandwich ELISA as previously described [27]. A 96-well plate was coated with goat polyclonal anti-human CXCL8 antibody generated in our laboratory [28], followed by blocking with phosphate buffered saline (PBS) containing 0.1% (w/v) casein and 0.05% (v/v) Tween 20. Total human CXCL8 in samples was detected by mouse monoclonal anti-human CXCL8 antibody (R&D Systems, Abingdon, UK) and by a secondary peroxidase-conjugated anti-mouse IgG antibody (Jackson ImmunoResearch Laboratories, West Grove, PA, USA). Peroxidase activity was quantified by measuring the conversion of 3,3′,5,5′-tetramethylbenzidine (TMB) (Sigma-Aldrich) at 450 nm.

### Detection of citrullinated CXCL8

**Figure 1:**
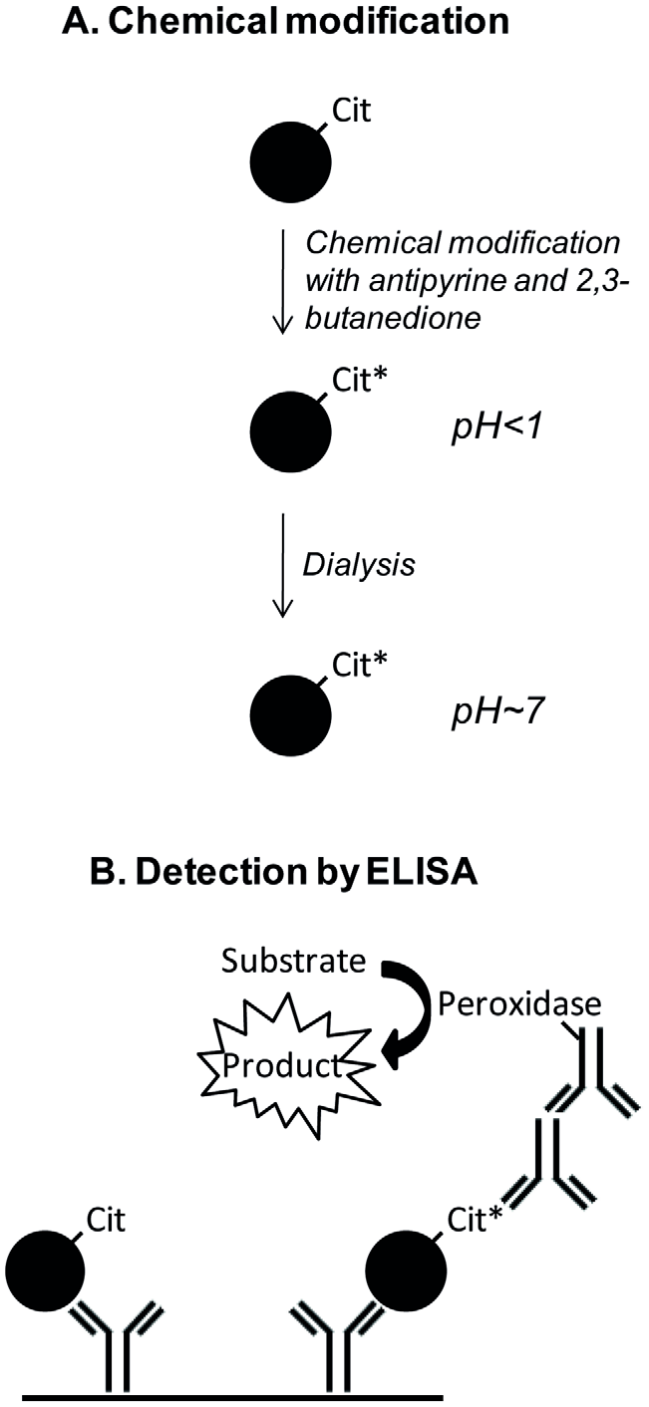
Principle of chemical modification and detection of citrulline-containing proteins. Peptidylcitrullines are chemically modified with antipyrine and 2,3-butanedione at low pH, followed by dialysis against a buffer with neutral pH (Panel A) and chemically modified citrullinated proteins are detected by sandwich ELISA (Panel B). Coating antibodies are specific for the protein of interest and antibodies against modified citrulline residues and peroxidase labelled anti-rabbit antibodies are used for detection. Chemically modified citrulline (Cit) is marked with an asterisk.

The procedure to chemically modify and detect a citrulline-containing protein of interest is shown in Figure 1. Peptidylcitrulline residues in samples were chemically modified by addition of 50 mM antipyrine, 16% TFA and 12.5 mM 2,3-butanedione [25] and incubation for 2 h at 37°C in the dark. The strongly acidic reaction mixture was dialyzed against PBS containing 0.05% Tween 20 (pH7.4) overnight at room temperature and protected from light using Slide-A-Lyzer®MINI Dialyse Units (Pierce).

To detect citrullinated CXCL8, a specific sandwich ELISA was developed. A 96-well plate was coated with mouse monoclonal anti-human CXCL8 antibody (R&D Systems), followed by blocking with PBS containing 0.1% casein and 0.05% Tween 20. Human [Cit^5^]CXCL8 in chemically modified samples was detected by the specific rabbit antibodies against chemically modified citrulline residues and by a secondary peroxidase-conjugated anti-rabbit IgG antibody (Jackson ImmunoResearch Laboratories) (Figure 1). Peroxidase activity was quantified by measuring the conversion of TMB at 450 nm.

### Cell cultures and induction experiments

PBMCs and granulocytes were isolated from human buffy coats (blood transfusion center of the Red Cross, Leuven, Belgium) as previously described [18], [29]. PBMCs and granulocytes were separated by density gradient centrifugation on Ficoll-sodium diatrizoate (Lymphoprep; Axis-Shield PoC AS, Oslo, Norway) for 30 min at 400 g. PBMCs and granulocytes were obtained from the supernatant and pellet, respectively. Erythrocytes in the granulocyte pellet were removed by sedimentation for 30 min at 37°C in hydroxyethyl-starch solution (Plasmasteril; Fresenius AG, Bad Homburg, Germany). Granulocytes were additionally subjected to a hypotonic shock for 30 seconds to remove remaining erythrocyte contamination and both PBMCs and granulocytes were washed with PBS. PBMCs and granulocytes were seeded at 2×10^6^ cells/ml and 10×10^6^ cells/ml, respectively, in 48-well dishes (500 µl/well) at 37°C and 5% CO_2_ in RPMI 1640 containing 2% (v/v) FBS and treated with LPS (0, 0.1, 1 and 10 µg/ml). Conditioned media were harvested after 24 h of incubation and kept at −20°C until further analysis.

## Results

### Chemical modification of peptidylcitrulline

**Figure 2:**
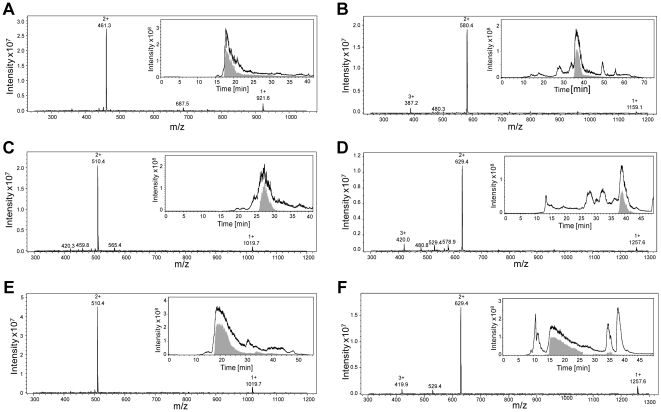
Ion trap mass spectra of purified synthetic peptides containing citrulline and chemically modified synthetic peptides. Peptides (YAGCitLLTK-NH_2_ [monoisotopic M_r_ = 920.5], PIECitTKLY-NH_2_ [monoisotopic M_r_ = 1018.6], and PIECitTYLK-NH_2_ [monoisotopic M_r_ = 1018.6]) were synthesized based on fluorenyl methoxycarbonyl (Fmoc) chemistry, purified using RP-HPLC, chemically modified with antipyrine and 2,3-butanedione and repurified by RP-HPLC. The M_r_ of the purified synthetic peptides was determined by deconvolution of the multiple charged ions in the raw spectra. As expected the M_r_ of the chemically modified synthetic peptides differs 238 mass units from the corresponding unmodified peptide. Total ion chromatograms (TIC; black lines) and extracted ion chromatograms for the charged ions of the specific peptides (EIC; gray fill) are shown as inserts in the experimentally determined mass spectra for (A) YAGCitLLTK-NH_2_ [M_r_ = 920.6], (B) YAGCit*LLTK-NH_2_ [M_r_ = 1158.6], (C) PIECitTKLY-NH_2_ [M_r_ = 1018.7], (D) PIECit*TKLY-NH_2_ [M_r_ = 1256.8], (E) PIECitTYLK-NH_2_ [M_r_ = 1018.7] and (F) PIECit*TYLK- NH_2_ [M_r_ = 1256.8]. Citrullines (Cit) marked with an asterisk are chemically modified.

**Figure 3:**
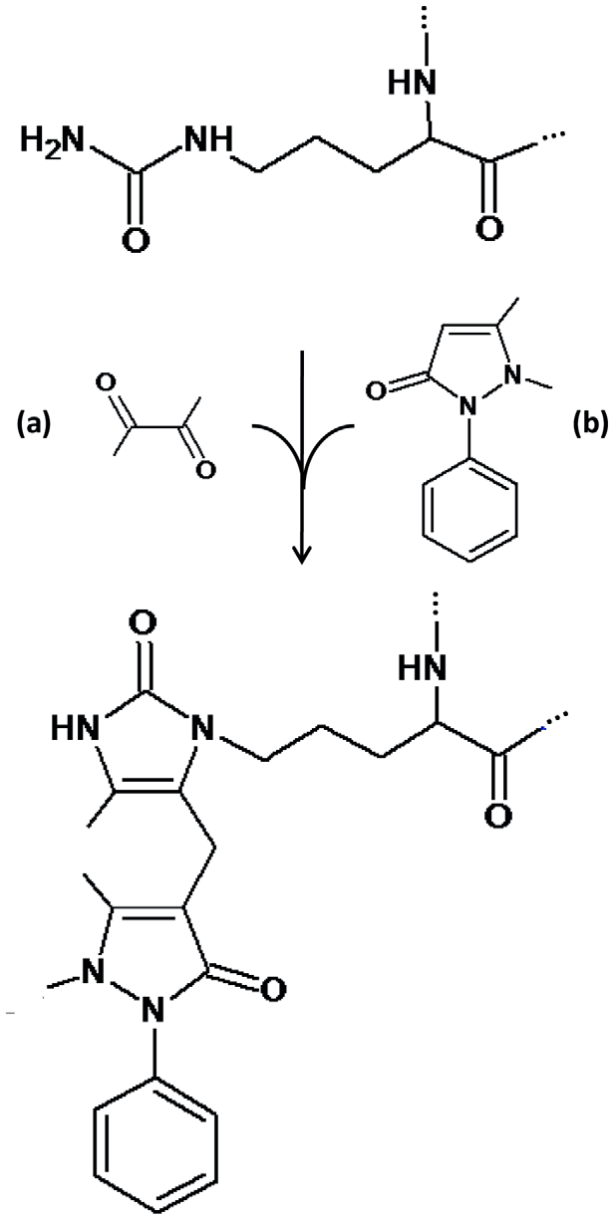
Chemical modification of the ureido group of a citrulline residue by 2,3-butanedione and antipyrine. First 2,3-butanedione (a) reacts with the ureido group of citrulline to form a reactive imidazolone derivate (M_r_+50). The first part of the reaction is followed by a nucleophilic addition of antipyrine (b) (M_r_+188) to the imidazolone ring. The full reaction results in a total mass shift of +238Da.

Three synthetic peptides presented in Table 1 were synthesized based on Fmoc chemistry, deprotected, purified by RP-HPLC and analyzed by online ion trap mass spectrometry (Figure 2). Subsequently, the peptides were chemically modified using antipyrine and 2,3-butanedione. Ion trap mass spectrometry data showed that the modification reaction and purification resulted in a homogeneous product with a mass increase of 238 mass units for all three peptides (Figure 2 and Table 1). The observed mass shift of +238 corresponds to a chemical reaction of 2,3-butanedione with the ureido group of citrulline to form a reactive imidazolone derivate (M_r_+50), followed by a nucleophilic addition of antipyrine (M_r_+188) to the imidazolone ring (Figure 3). Under the applied acidic conditions this reaction mechanism is specific for citrulline residues [25], [26].

**Table 1:** Sequence and M_r_ of synthetic citrulline-containing peptides, before and after chemical modification.

Synthetic peptide	Sequence	Theor. M_r_ a	Exp. det. M_r_ b
***Pept1***	YAGCitLLTK-NH_2_	920.5	920.6
***Pept2***	PIECitTKLY-NH_2_	1018.6	1018.7
***Pept3***	PIECitTYLK-NH_2_	1018.6	1018.7
***Modified pept1*** c	YAGCit*LLTK-NH_2_	1158.5	1158.6
***Modified pept2*** c	PIECit*TKLY-NH_2_	1256.6	1256.8
***Modified pept3*** c	PIECit*TYLK-NH_2_	1256.6	1256.8

a Theoretical monoisotopic M_r_.

b Experimentally determined M_r_.

c Cit* indicates that this citrulline was chemically modified with antipyrine and 2,3-butanedione.

### Specificity of the developed antibodies for chemically modified peptidylcitrulline

**Figure 4:**
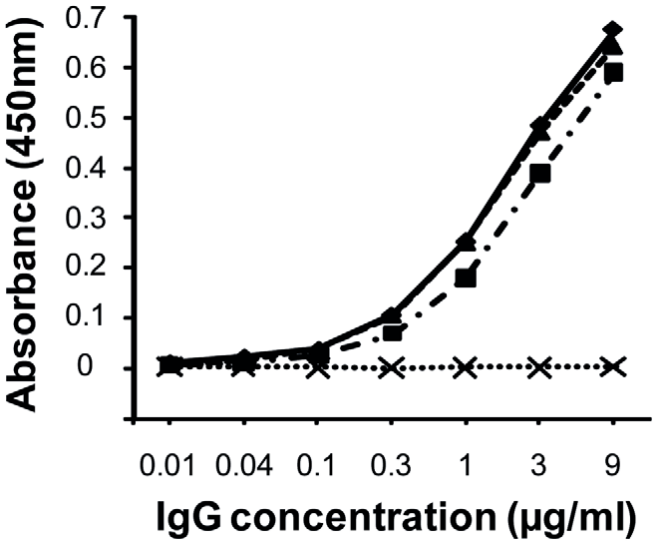
Antibody specificity for modified peptidylcitrulline. Binding of the antibody (absorbance at 450 nm) with 3 peptides containing modified citrulline [YAGCit*LLTK-NH_2_ (♦, full line), PIECit*TKLY-NH_2_ (▪, dotted and dashed line) and PIECit*TYLK-NH_2_ (▴, dashed line)] and the control unmodified peptide YAGCitLLTK-NH_2_ (x, dotted line) was measured by ELISA. Citrullines (Cit) marked with an asterisk are chemically modified.

A New Zealand white rabbit (*Oryctolagus cuniculus*) was immunized with chemically modified peptide1 YAGCit*LLTK-NH_2_ coupled to KLH. Antibodies against chemically modified citrulline residues were purified from the serum by affinity chromatography on Protein G. The specificity of antibodies for modified peptidylcitrulline was evaluated by ELISA through binding of the purified antibody to plates coated with either of the 3 peptides containing modified citrulline (YAGCit*LLTK-NH_2_, PIECit*TKLY-NH_2_ and PIECit*TYLK-NH_2_) or the control unmodified peptide1 (YAGCitLLTK-NH_2_) (Figure 4). The antibody reacted with all three chemically modified citrullinated peptides but not with the unmodified control peptide. Thus, the polyclonal antibodies were highly specific for the chemical group of 238 mass units attached to citrulline after chemical modification and do not react with the backbone peptide structure.

### Specificity and sensitivity of the antibody-based detection method

In order to detect citrullinated chemokines specifically, proteins were first chemically modified with antipyrine and 2,3-butanedione at low pH. The chemical modification step was followed by dialysis against a buffer with neutral pH. 96-well plates were coated with anti-chemokine antibodies and bound modified chemokine was detected using the antibodies against the modified citrulline residues (Figure 1).

**Figure 5:**
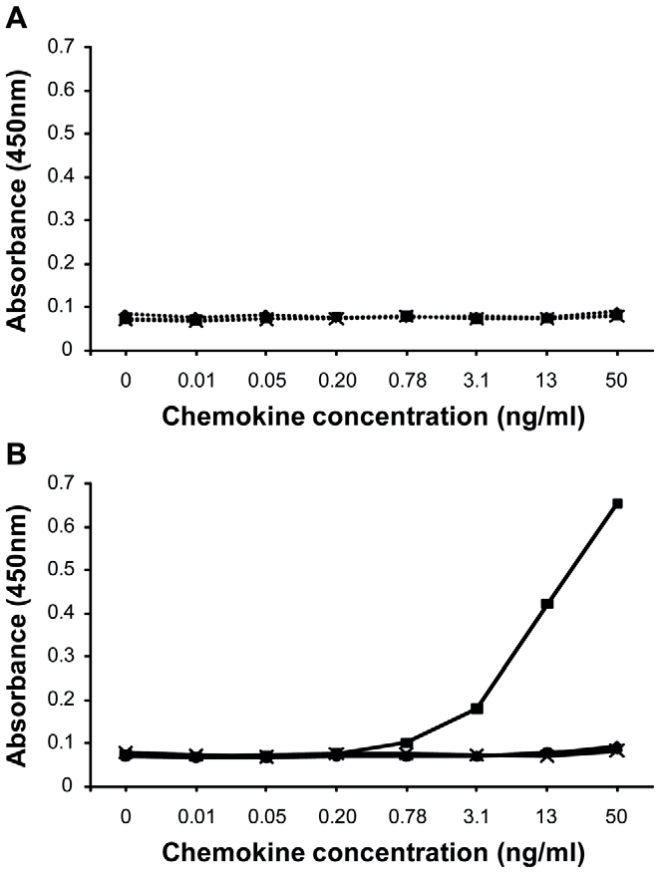
Specificity of the ELISA for chemically modified citrullinated CXCL8. Chemokines [CXCL8 (♦)], citrullinated chemokines [[Cit^5^]CXCL8 (▪) and [Cit^9^]CXCL5 (•)] and RPMI 1640 medium containing 2% FBS [control medium (x)] were chemically modified with antipyrine and 2,3-butanedione at low pH. Subsequently, samples were dialyzed against a buffer with neutral pH and modified citrullinated CXCL8 ([Cit^5^]CXCL8*) was detected by ELISA using anti-CXCL8 coating antibodies and detection antibodies against modified citrulline residues. Untreated samples (Panel A) and samples treated with antipyrine and 2,3-butanedione (Panel B) are represented as dotted and full lines respectively.

**Figure 6:**
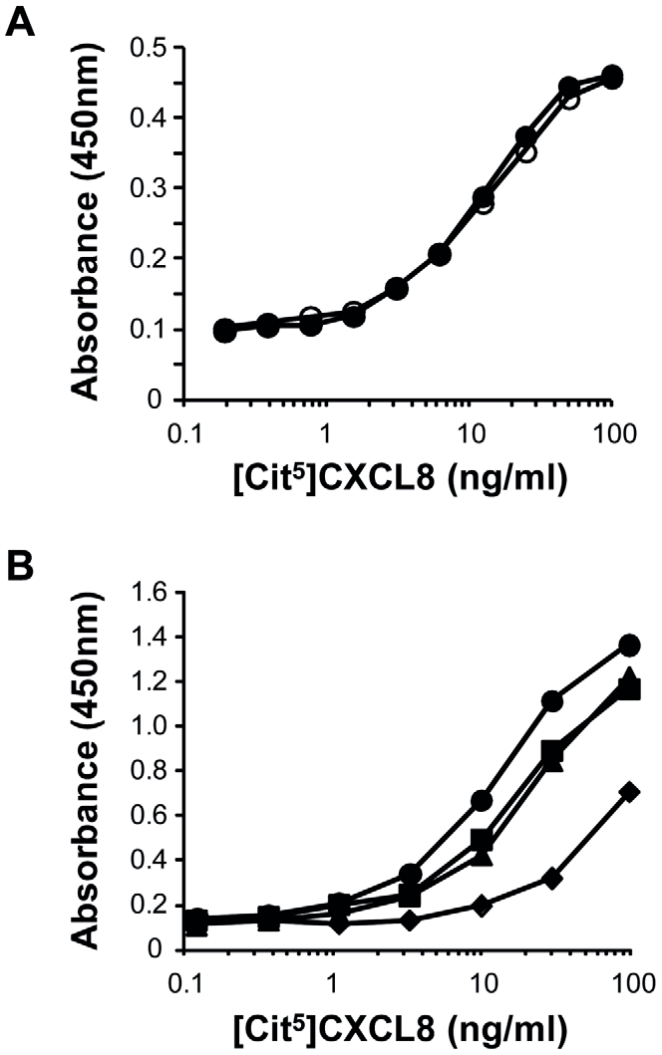
ELISA standard curves for citrullinated CXCL8 in various biological fluids. Standard curves for [Cit^5^]CXCL8 were generated in culture medium containing 10% FBS or in human plasma, serum or whole blood. The indicated concentrations of [Cit^5^]CXCL8 represent the initial concentrations spiked in the complex samples before chemical modification and dialysis. Panel A shows two independent standard curves for [Cit^5^]CXCL8, standard 1 (○) and 2 (•), in RPMI 1640 medium containing 10% FBS. For both standards the detection range is 1–50 ng/ml of [Cit^5^]CXCL8 in the original sample. In a second series of experiments (Panel B) standard curves for [Cit^5^]CXCL8 were generated in RPMI 1640 medium containing 10% FBS (•) or in human plasma (▪), serum (▴) or whole blood (♦) (average of two independent experiments).

To test the specificity of the two-step procedure, the defined citrulline-containing chemokine, i.e. [Cit^5^]CXCL8, was selected as a model protein. Intact CXCL8 and the citrullinated chemokines [Cit^5^]CXCL8 and [Cit^9^]CXCL5, as well as cell culture medium were chemically treated with antipyrine and 2,3-butanedione at low pH, followed by dialysis against PBS with neutral pH and detected by ELISA using a monoclonal antibody against CXCL8 as coating antibody and a polyclonal antibody against modified citrulline residues for detection. After chemical modification of the samples the absorbance increased proportional with the concentration of [Cit^5^]CXCL8 in the sample (Figure 5, Panel B). [Cit^9^]CXCL5, non-citrullinated CXCL8 and the cell culture medium were not detected. When no chemical modification was introduced, none of the tested proteins including [Cit^5^]CXCL8, were detected (Figure 5, Panel A). When RPMI 1640 culture medium with 10% FBS was spiked with [Cit^5^]CXCL8, reproducible chemokine standard curves could be obtained with the newly developed method (Figure 6, Panel A). Chemically modified [Cit^5^]CXCL8 concentrations as low as 1 ng/ml could be measured in FBS-containing samples. To test the detection of [Cit^5^]CXCL8 in clinically relevant samples, chemokine standards were generated in human plasma, serum and whole blood (Figure 6, Panel B). After spiking with [Cit^5^]CXCL8, whole blood was centrifuged for 10 min at 240 g and 10 min at 7800 g to obtain platelet-free plasma prior to the chemical treatment with antipyrine and 2,3-butanedione. Comparable standard curves were obtained for [Cit^5^]CXCL8 in medium containing FBS, in human plasma or serum. However, when 5 ng [Cit^5^]CXCL8 was spiked in 50 µl whole human blood, the recovery rate was significantly lower. This is not surprising in view of the chemokine-binding receptor DARC present on the abundant amount of red blood cells in whole blood.

To further investigate whether citrullinated proteins could be fully recovered in complex mixtures, cell culture supernatants from PBMCs and granulocytes were harvested and CXCL8 concentrations were determined. Afterwards these supernatants were spiked with [Cit^5^]CXCL8, chemically modified and [Cit^5^]CXCL8 levels measured. The percentage of [Cit^5^]CXCL8 recovery was calculated as shown in Table 2. In supernatants from PBMCs and granulocytes 84.0±10.8 and 87.1±11.5% of the spiked [Cit^5^]CXCL8 amount could be recovered.

**Table 2:** Chemically modified [Cit^5^]CXCL8 recovery from cell culture supernatants.

Complex sample	[Cit^5^]CXCL8 recovery (%) ± SEM
*PBMCs*	84.0±10.8
*Granulocytes*	87.1±11.5

Culture supernatants of unstimulated cells were spiked with 20 ng/ml [Cit^5^]CXCL8 before chemical modification and detection. Results represent the mean % (± SEM) for n = 11.

### Effect of LPS on the citrullination of CXCL8 produced by leukocytes

**Figure 7:**
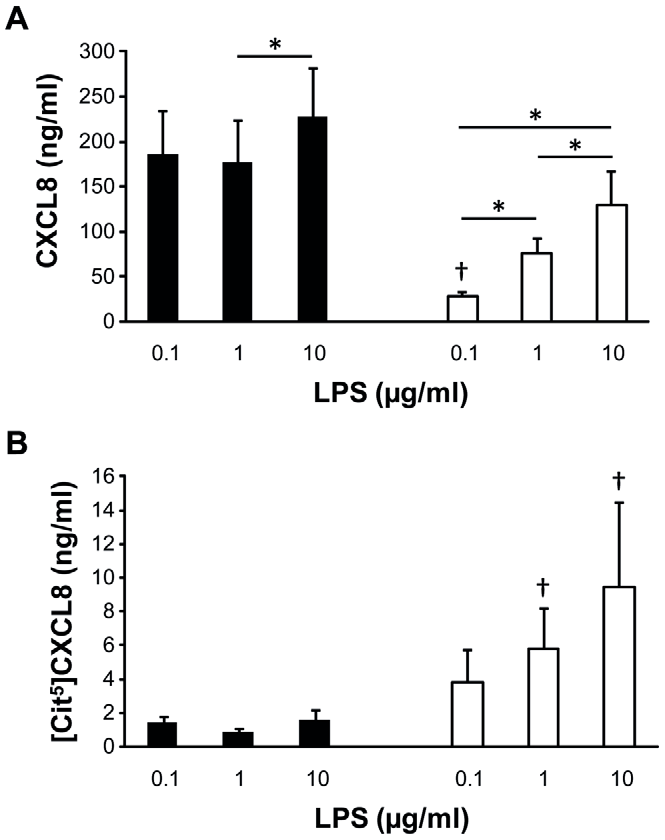
Effect of LPS on the induction of total CXCL8 and [Cit^5^]CXCL8 in PBMCs and granulocytes. PBMCs (black) and granulocytes (white) were induced with 0, 0.1, 1 and 10 µg/ml LPS and cell culture supernatants harvested after 24 h. CXCL8 and [Cit^5^]CXCL8 concentrations were measured by ELISA and the newly developed antibody-based method, respectively. Results represent the mean concentration of CXCL8 (Panel A) and [Cit^5^]CXCL8 (Panel B) in ng/ml (seven independent experiments on cells from different donors). Statistical comparison between production levels of CXCL8 and [Cit^5^]CXCL8 was performed using the Wilcoxon matched pairs test (* p<0.05 for comparison within a leukocyte subgroup and † p<0.05 for comparison with PBMCs induced with the same dose of LPS).

PBMCs and granulocytes were induced with 0, 0.1, 1 and 10 µg/ml LPS. After 24 h of incubation CXCL8 concentrations were measured. Without LPS stimulation the amount of CXCL8 produced by PBMCs or granulocytes was below the detection limit of the CXCL8 ELISA. As shown in Figure 7 (Panel A), levels of CXCL8 produced by granulocytes (10×10^6^ cells/ml) increased significantly when more LPS was added. Maximal CXCL8 production was already reached when PBMCs (2×10^6^ cells/ml) were treated with 0.1 µg/ml LPS. Compared to PBMCs, granulocytes secreted significantly less CXCL8 in the presence of 0.1 µg/ml LPS. Subsequently, the [Cit^5^]CXCL8 concentration in the supernatants was determined after chemical modification (Figure 7, Panel B). In conditioned medium of LPS-treated PBMCs almost no CXCL8 citrullination occurred. In contrast, granulocytes produced significantly higher amounts of [Cit^5^]CXCL8.

## Discussion

PTM have an important impact on the biological activity of cytokines and chemokines [6], [7]. Chemokine quantification in biological samples, based on standard ELISAs with chemokine specific monoclonal antibodies, is very selective for an individual gene product of interest, but in most cases does not allow discrimination between different posttranslationally modified forms of the same protein [30]. To further investigate the biological importance of PTM the development of techniques able to distinguish between posttranslationally modified proteins is necessary.

A recently discovered PTM on chemokines, i.e. deimination or citrullination, regulates the biological function of these proteins and may also be involved in the development of diverse pathologies like rheumatoid arthritis and multiple sclerosis [12]. Citrulline is not one of the 20 natural amino acids encoded by DNA but is the result of a PTM induced by PADs. If proteins are citrullinated under pathological conditions, the novel epitope may induce an autoimmune response. As such, anti-citrullinated protein antibodies (ACPA) are the best diagnostic marker for rheumatoid arthritis to date [31]. Moreover, the degradation of myelin in the central nervous system is the hallmark of multiple sclerosis. Reduction of the net positive charge of myelin basic protein (MBP), through deimination, correlates strongly with disease severity and may mediate myelin instability and loss of compaction [15]. Therefore, it's important to detect and quantify citrullinated proteins in biological samples. However, citrullination results in an increase of only one mass unit and the loss of one positive charge. Such minor change in M_r_ is difficult to detect by mass spectrometry. Furthermore, antibodies against a specific citrullinated protein, like citrullinated antithrombin [32], were generated. The disadvantage of such antibodies specific for one protein is that for each application the production of novel antibodies is needed. Moreover, we experienced in case of CXCL8 that monoclonal antibodies generated against a citrullinated protein do not necessarily discriminate between unmodified and citrullinated isoforms (data not shown). Recently, a chemical method was described to chemically modify citrulline residues in proteins. Indeed, antipyrine and 2,3-butanedione at low pH specifically react with citrulline resulting in a modification of 238Da [25]. This allows for the identification of citrullinated proteins by mass spectrometry [26]. However, quantification of citrullinated proteins by mass spectrometry in biological samples is time consuming and requires the use of complex and expensive techniques not available in most standard laboratories. Therefore, we developed an immunological detection method for citrullinated proteins based on antibodies raised against chemically modified citrulline residues. This method should allow detecting and quantifying any citrullinated protein of choice by changing the coating antibody used in the ELISA.

The above procedure was validated for [Cit^5^]CXCL8 as a model chemokine. Indeed only chemically modified citrullinated CXCL8 was detected and reproducible standard curves ranging from 1 to 50 ng/ml [Cit^5^]CXCL8 were successfully generated in culture medium containing 10% FBS, in human plasma, serum and whole blood. The high specificity and selectivity of our method for [Cit^5^]CXCL8 is most likely explained by the high specificity of the modification reaction for citrulline under the strong acidic conditions used [25] and by the selective binding of the generated antibody against the chemically modified citrulline. It was already reported that median CXCL8 levels in synovial fluid from patients suffering from autoimmune arthritis vary between 1–10 ng/ml [33]. Also in cell conditioned media containing an excess of background protein, [Cit^5^]CXCL8 could successfully be detected by the method described here with a recovery of about 85%. The newly developed method to specifically detect citrullinated proteins was also used to investigate the influence of LPS on the citrullination of CXCL8 induced in PBMCs and granulocytes. Human monocytes and neutrophils produce CXCL8 upon stimulation with LPS [20], [34], [35]. PAD are the enzymes responsible for the citrullination of proteins. The isotype PAD4 is mainly expressed in white blood cells like granulocytes and monocytes [36], [37] and can citrullinate CXCL8 [8]. Comparison of different leukocyte subpopulations revealed significantly higher [Cit^5^]CXCL8 levels in supernatants of granulocytes in comparison to PBMCs. Neutrophils play an essential role in innate immunity, being the first cells recruited to the site of infection [38]. Neutrophils can trap and kill various bacterial, fungal and protozoal pathogens by the formation of chromatin structures loaded with antimicrobial molecules, known as neutrophil extracellular traps (NET) [39]. Upon stimulation with inflammatory stimuli like LPS, the activated neutrophils will generate the extracellular traps. Citrullination of proteins like histones, mediates NET formation as first line of defense against pathogens [40] and PAD4 is essential for NET-mediated antibacterial function [41]. Our results might suggest that PAD4 in these NETs not only citrullinates histones, but also non-structural immunomodulatory proteins such as CXCL8. Citrullination dampens the inflammatory properties of CXCL8 [8], [18] and possibly citrullination is used as a mechanism to control the immune response.

In conclusion, a newly developed antibody-based method to specifically detect and quantify chemically modified citrullinated proteins is proven to be effective. This study furthermore demonstrates that stimulated granulocytes produced significant levels of [Cit^5^]CXCL8.
